# Advancing the application of systems thinking in health: why cure crowds out prevention

**DOI:** 10.1186/1478-4505-12-28

**Published:** 2014-06-16

**Authors:** David Bishai, Ligia Paina, Qingfeng Li, David H Peters, Adnan A Hyder

**Affiliations:** 1 Department of Population Family and Reproductive Health, Johns Hopkins Bloomberg School of Public Health, 615 N. Wolfe St., Room E4622, Baltimore, MD 21205, USA; 2 Department of International Health, Johns Hopkins Bloomberg School of Public Health, 615 N. Wolfe St., Baltimore, MD 21205, USA; 3 International Injury Research Unit, Johns Hopkins Bloomberg School of Public Health, 615 N. Wolfe St., Baltimore, MD 21205, USA

## Abstract

**Introduction:**

This paper presents a system dynamics computer simulation model to illustrate unintended consequences of apparently rational allocations to curative and preventive services.

**Methods:**

A modeled population is subject to only two diseases. Disease A is a curable disease that can be shortened by curative care. Disease B is an instantly fatal but preventable disease. Curative care workers are financed by public spending and private fees to cure disease A. Non-personal, preventive services are delivered by public health workers supported solely by public spending to prevent disease B. Each type of worker tries to tilt the balance of government spending towards their interests. Their influence on the government is proportional to their accumulated revenue.

**Results:**

The model demonstrates effects on lost disability-adjusted life years and costs over the course of several epidemics of each disease. Policy interventions are tested including: i) an outside donor rationally donates extra money to each type of disease precisely in proportion to the size of epidemics of each disease; ii) lobbying is eliminated; iii) fees for personal health services are eliminated; iv) the government continually rebalances the funding for prevention by ring-fencing it to protect it from lobbying.

The model exhibits a “spend more get less” equilibrium in which higher revenue by the curative sector is used to influence government allocations away from prevention towards cure. Spending more on curing disease A leads paradoxically to a higher overall disease burden of unprevented cases of disease B. This paradoxical behavior of the model can be stopped by eliminating lobbying, eliminating fees for curative services, and ring-fencing public health funding.

**Conclusions:**

We have created an artificial system as a laboratory to gain insights about the trade-offs between curative and preventive health allocations, and the effect of indicative policy interventions. The underlying dynamics of this artificial system resemble features of modern health systems where a self-perpetuating industry has grown up around disease-specific curative programs like HIV/AIDS or malaria. The model shows how the growth of curative care services can crowd both fiscal and policy space for the practice of population level prevention work, requiring dramatic interventions to overcome these trends.

## Introduction

Achieving optimum health of a population requires an artful combination of preventing ill health and responding to disease cases with curative services. Both are important, but there are predictable obstacles to achieving balance. Too often, only a single, limited government health budget is available for investments in both non-personal preventive and curative personal health services. A variety of influences affect the allocation of this common health budget [1]. Ideally, these factors include efficiency and equity. In reality, concerns also include policy and political priorities, which often take precedence over efficiency and equity criteria [2]. This struggle between evidence-based decision-making to achieve health system goals and the reality of policy and financing constraints occurs in a variety of contexts besides government health ministries.

Fixed government health budgets lend themselves to a zero-sum game in resource allocation between cure and prevention. More spending on curing diseases will mean less for prevention and vice versa. It is observed with regularity, in both high-income and low- and middle-income settings, that whenever there is a fixed sum to be allocated between curing and preventing diseases, a higher total will be spent on curing than preventing, and more will be spent per disability-adjusted life year (DALY) averted by curing than preventing [3-6]. For example, although the burden of disease associated with chronic, non-communicable diseases is significant, in the Organization for Economic Co-operation and Development countries average expenditure on public health and prevention for non-communicable diseases was only 3% of the total health expenditure in 2005, while average expenditure on curative care was 57% [7]. The situation can be even direr in developing countries, particularly in sub-Saharan Africa, where the large urban hospitals often receive at least half of the public funds spent on health [8].

Spending more money per DALY averted on curing than preventing violates both efficiency and equity goals. It violates efficiency standards by definition. If intervention P saves more lives per incremental dollar than intervention C then a shift of spending from C to P will save more lives but it will not cost more. It also violates equity standards because access to curative services is often achieved preferentially by those with greater social privilege [9]. The preventive interventions we consider here are delivered to a population en masse rather than in individual clinical encounters and they have been shown to decrease population health disparities and increase health equity [10,11].

Preventing is not always more efficient than curing. Many preventive health care procedures delivered to individuals in clinics are not uniformly more cost-effective than curative clinical services [12]. However, most preventive interventions are not clinical procedures, they are community and environmental interventions mounted by public health entities. By shifting the environmental and social determinants of health for populations millions at a time, public health expenditures are typically best buys in health [13].

This paper analyzes a process whereby a neutral policy change undertaken in the name of efficiency can lead to a spiraling increase in the power of groups whose self-interest will block rational and efficient allocation of public resources in the future. A standard decision analysis of option A vs. option B will be inadequate if option A commits future generations to depart from rational policy making because of the power of interest groups created by option A. To be specific, the model developed here examines how health policies may enhance the class power of curative care interests (e.g., clinicians, hospitals, medicine manufacturers) and lead to a snowball effect that exaggerates a bias to spend more towards clinically-oriented health spending in the future.

Power politics are unavoidable in health policy [14]. Examples of policy makers successfully appealing to cost-effectiveness data and not politics to rationalize their health spending portfolio are few [15]. In fact, many examples show that policy-makers do not use cost-effectiveness data to decide on budget allocation [16,17]. There are simple explanations for why decision-makers whose stated objective in budgetary allocation is to avert DALYs at lowest cost fail to actually allocate spending accordingly. Most explanations focus on the decision-makers lack of cost-effectiveness data or unfamiliarity with the paradigm [2,12]. However, the regularity of the bias towards cure and away from prevention suggests that something more structural and systematic must be at work.

The models that will yield understanding will need to encompass unintended consequences of complex adaptive systems. A growing body of literature explores the role of complexity in health systems [18-21]. For this paper, we use the principles of system dynamics modeling to develop understanding of non-linear interactions in defined systems [22]. Using system dynamics, researchers can simulate policy scenarios which cannot be carried out in real populations or for which adequate historical data on natural experiments is not available. We offer a simple model of political lobbying between the curative health care sector and public health proponents situated in a hypothetical population with a very simple epidemiological problem.

We are pursuing a form of “generative” social science—applying the adage “*if you didn’t grow it, you didn’t explain it*” [23]; being able to generate a phenomenon without pre-supposing it is the best way to understand it, and this is best done in a simulation. The model is simple enough so that we can turn parts on and off allowing readers to understand which of the dynamics emerge from the simplistic assumptions and which result from the policy experiments. The omitted complexities of real world spending and lobbying would certainly mitigate the dire consequences that befall the population in our simplified model. That underscores the advantage of the model in offering insight into processes that are harder to measure in the thicket of real world observations. The model intentionally exaggerates important aspects of the real system – the exaggeration is a feature, not a flaw.

This artificial model is not trying to fit any real-world epidemiological data. The purpose of the model is to gain understanding of elementary political forces that can be turned on and off in the model. Specifically, one can test the effect on population health of government policies that:

i) Rationally allocate spending according to neutral cost-effectiveness criteria.

ii) Allocate government funds proportionally to interest group lobbying or not.

iii) Accept or refuse spending by non-governmental organization (NGO) external donors that target either disease A or B.

iv) Allow fee-for-service revenue for curative care workers.

## Methods

We model a finite population that is susceptible to only two possible unwanted health conditions. Individuals can suffer from an acute disease, called disease A, that is disabling, never lethal, and whose duration can be shortened by a visit to a doctor. They can also suffer from a sudden, instantaneously lethal condition, disease B, that can never be cured, but which can be prevented through environmental engineering by preventive care workers (PCWs) (if it helps, one can think of disease A as something like an intestinal parasite, e.g., ascariasis, and disease B as something like a bicycle crash). Disease A, if untreated, causes 100% disability for 0.5 years before recovery. Each untreated case of disease A imposes 0.5 DALYs on the population. Disease B kills each victim instantly and deprives them of 25 additional years of survival; each case of disease B imposes 25 DALYS. Both the doctors and the PCWs are supported by a fixed-sum budget allocated by the government. In contrast to the PCWs, the doctors can also collect fees from each patient who sees them after contracting disease A. Both PCWs and doctors invest a similar portion of their earnings on lobbying the government for a larger share of the fixed-sum budget. The government can be swayed by the lobbying, in which case it allocates the budget proportionally to the size of the lobbying funds of the respective two parties, e.g., if doctors account for 2/3 of all of the money spent on lobbying, they will get 2/3 of the health budget.

In the baseline mode, initial values were selected to put the model at a stable equilibrium prior to the introduction of shocks to the system. The initial baseline equilibrium does not include any external funding, and there are no shifts in incidence of either disease. In the baseline, all inflow and outflow equations are balanced perfectly. Subsequently, the response of the model to policy shocks can be fully attributed to the policy changes. A stock-and-flow diagram and simulation model was created using the VensimPLE^©^ software [24]. Consistent with system dynamics methodology, there are three types of variables for each sub-system: state variables depicting levels, difference equations depicting flow rates, and auxiliary variables reflecting other parameters [22]. Our stock-and-flow model is comprised of the three subsystems described below.

The model was designed solely by the investigators during a number of team meetings by DB, LP, and QL to redraw and recalibrate the feedback loops and adjust the parameters based on model output. Some studies in systems science are done with a specific decision-maker or institutional client in mind. In these cases, it is quite common to involve those decision-makers in helping to design the model. This engages the community of practice and research together and helps the group interpret the output of the model to jointly improve organizational policy. However, a drawback of this bespoke approach is that the better the model fits a specific problem, the less it fits a general problem. The research here is intended to be of general relevance to any setting where there is a zero sum budget that could be allocated to prevention or cure.

### Subsystem 1: the population and disease model

**Figure 1:**
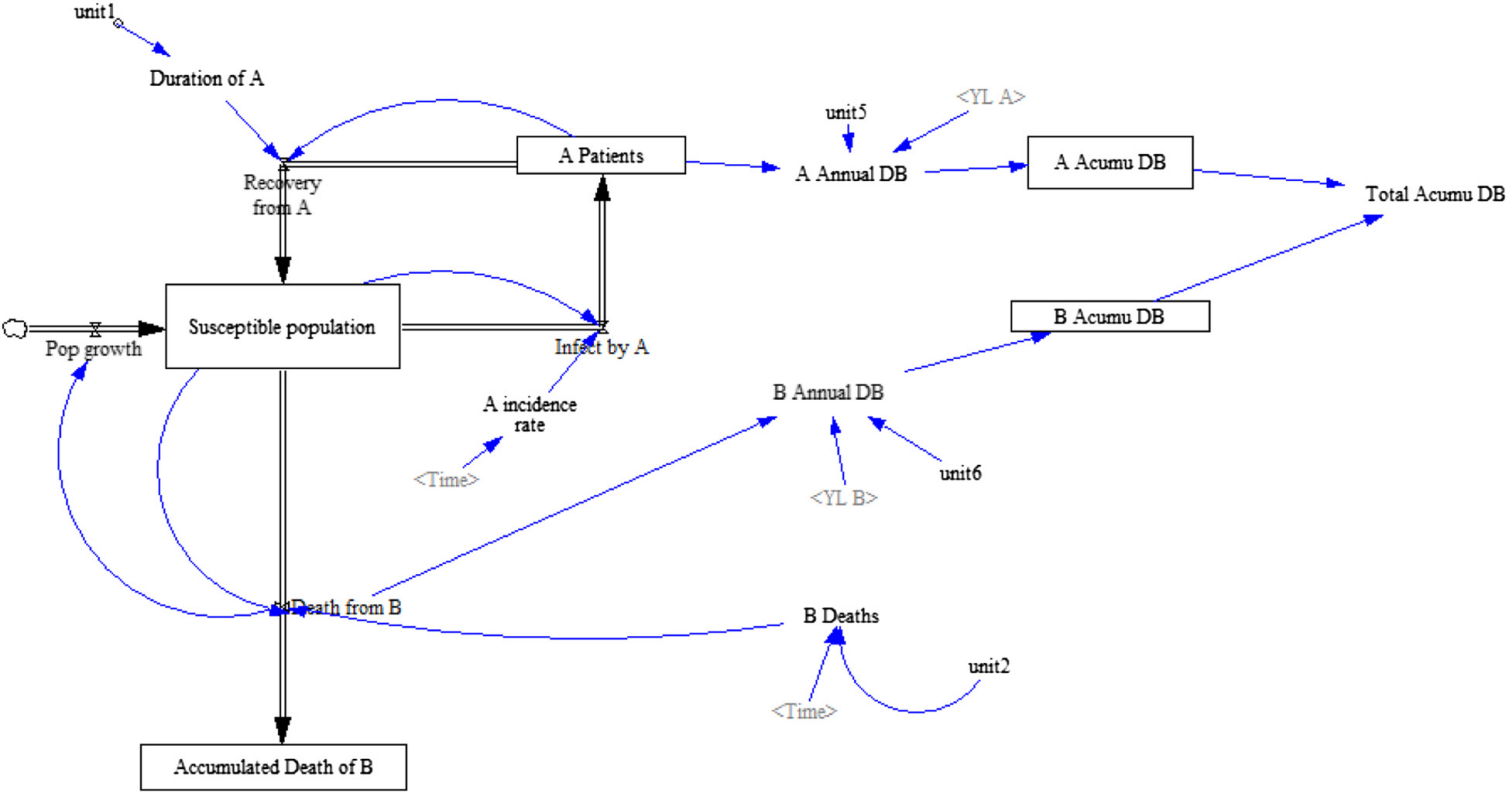
**Subsystem 1: the population model.** A susceptible population is at risk for either dying from disease B or transitioning temporarily into disease A. Abbreviations: A Acumu DB, Accumulated DALYS from A; B Acumu DB, Accumulated DALYs from B; Total Accumu DB, Total accumulated DALYs.

The subsystem for the population and its experience of diseases A and B can be found in Figure 1. Table 1 presents the population parameters, initial values, and relevant assumptions. There is a stable healthy population in which a person stops being healthy either temporarily if an individual contracts disease A, from which each will recover after a certain duration determined by the activity of doctors. Population members can also exit permanently by sudden death from disease B. If they do not die of disease B, individuals will all die 25 years later through a process unspecified by the model. The population count remains stable because fertility is unimportant to the focus of the model. All deaths are immediately replaced by new healthy full grown individuals with remaining lifespans of 25 years. The duration of disease A is influenced by medical care expenditures by government (and donors) and by fees paid to doctors in exchange for curative services. Both the acquisition of disease A and death from disease B lead to increments in the DALYS lost by the population. Hence, the relative impact of one event of either condition on the DALY measure of population health is just a matter of the arbitrary DALY weights. In the baseline model, we set these weights so that the population experiences an equal burden of DALYS from diseases A and B.

**Table 1:** Population subsystem parameters and assumptions

**Equilibrium parameters**	**Variable type**	**Initial value**	**Notes and assumptions**
Susceptible population	State	800	Individuals
Disease A infection rate	Rate	40	Individual infected/year
Disease A patients	State	200	Infected population, at equilibrium
Disease A incidence rate	Auxiliary	0.05	Population based incidence
Disease A duration	Auxiliary	0.2	Years
Disease A recovery rate	Rate	40	All infected with disease A, recover
Death from disease B	Rate	4	Deaths/year
Accumulated death from disease B	State	0	Deaths from B, at equilibrium
Disease A annual burden	Auxiliary	100	Disease A burden at equilibrium
DALYs lost per case of disease A	Auxiliary	0.5	Constant – one untreated case of infection with A is equivalent to 0.5 years of life with disability
Disease A cumulative burden	State	0	Disease A burden at equilibrium
Disease B annual burden	Auxiliary	100	Disease B burden at equilibrium
DALYs lost per case of disease B	Auxiliary	25	Constant – one death from B is equivalent to 25 years of life lost
Disease B cumulative burden	State	0	Disease B burden at equilibrium
Total cumulative disease burden	Auxiliary	0	Sum of cumulative burden for disease A and disease B, at equilibrium

The fundamental difference between the curable disease A and the preventable disease B in the model is not the DALY weights assigned to each as later shown in the sensitivity analysis. The key asymmetry between A and B is that there is no market for PCWs to charge fees for preventing disease B. Epidemics of curable diseases will fill the doctor’s offices with paying customers, but an epidemic of disease B cannot generate a revenue surge in the absence of government action. This difference in the model corresponds to a difference in real health policy, especially remembering that the “prevention” that is being modeled is not clinical preventive services which can generate revenue, but community level public health activities (e.g., road hazard reduction) for which revenue collection is necessarily collective. Even though real world doctors have higher incomes than PCWs, we set their baseline income to be equal in the model. The incomes become unbalanced only when we allow the doctors to charge fees to people with disease A. The assumption of equal baseline incomes helps us see exactly what is responsible for the power imbalances that will emerge. The power imbalance between cure and prevention is not assumed – the imbalance comes from policies that tie earning to curing.

### Subsystem 2: health resources

**Figure 2:**
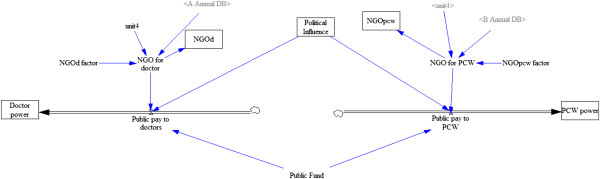
**Subsystem 2: doctors (left) and PCWs (right) accumulate “power” in the form of earnings.** A public fund (center bottom) is allocated to public payments to doctors (arrow pointing left) and public payments to PCWs (arrow pointing right). There are also two non-governmental organizations (NGOs) named NGO-D, which donates to doctors, and NGO-P, which donates to PCWs. Consultation fees (bottom left) in proportion to the number of patients with disease A also supplement doctor’s earnings.

The subsystem depicting health resources and how they are allocated is illustrated in Figure 2. Table 2 presents parameters, their units, and any assumptions that are relevant for our design. The simulated health system has three principal funding sources: public funding for both PCWs and doctors, private spending for curative services only, and donor funding. Public funding is a finite source which is allocated between doctors and PCWs through a political bargaining process between the two categories of health workers vying for a finite pot of governmental health funds. Bargaining power is measured in monetary units and each group acquires “power” by paying a portion of their earnings into a bargaining fund. In a simple model of “rent-seeking” the government is influenced to allocate state resources to either group in proportion to their share of total bargaining power. Thus, allocation of the fixed health budget to C is proportional to C/(C + P) and allocation to P follows P/(C + P) where C is lobbying dollars spent by curative care interests and P is lobbying dollars spent by preventive care interests.

**Table 2:** Subsystem 2 variables and assumptions

**Equilibrium parameters**	**Variable type**	**Initial value**	**Notes and assumptions**
Public fund	Auxiliary	1250	Monetary units available, at equilibrium
Political influence	State	N/A	(Doctors spending on lobbying)/(Doctors spending on lobbying + PCWs spending on lobbying)
Public pay to doctors	Rate	N/A	(Political Influence*Public Fund) + NGO for doctor
Private pay for doctors	Rate	N/A	
Consultation fees for doctors	Auxiliary	3.75	Monetary units per treatment provided by doctor
Public pay to PCWs	Rate	N/A	Public Fund × (1–Political Influence) + NGO for PCW
NGO-D	State	0	Accumulated external funding to doctors
NGO-P	State	0	Accumulated external funding to PCWs

The model sets initial values such that the baseline government spending is economically optimal. At baseline, the dollars the government spends per DALY averted is exactly the same between diseases A and B. Emotional or political factors that might bias spending towards cure are not burnt in as an official part of government strategy. In addition to public funding, we set initial values so that the arrival of external aid from donors is always unbiased making the donor allocate new dollars to disease A or disease B to keep the incremental DALYS averted per dollar equal and unbiased. Such a policy is crudely feasible for donors today who could consult league tables that display comparative $ per DALY averted from various interventions across world regions [13,25]. In the baseline version of the model, these efficient spending levels are maintained in perpetuity because there are no epidemics that might trigger donor allocations or patient care seeking. Lobbying power and funding allocations between doctors and PCWs will remain perfectly balanced as long as incidence rates of either disease are unperturbed.

For policy simulation, the model imposes an exogenous series of epidemics of A and B as a series of step functions that raise the incidence of diseases A or B or both above the equilibrium every 3 to 4 years. The model is able to restore equilibrium after these step function epidemics because the government and/or donors immediately detect the epidemic and they rationally increase funding towards whatever disease has risen above its baseline. The government and donors are programmed to respond without bias to disease A or B. They are set to allocate the same $ per DALY averted to disease A as is allocated to disease B during an epidemic, as long as there is no political lobbying.

### Subsystem 3: doctor and PCW resource allocation

**Figure 3:**
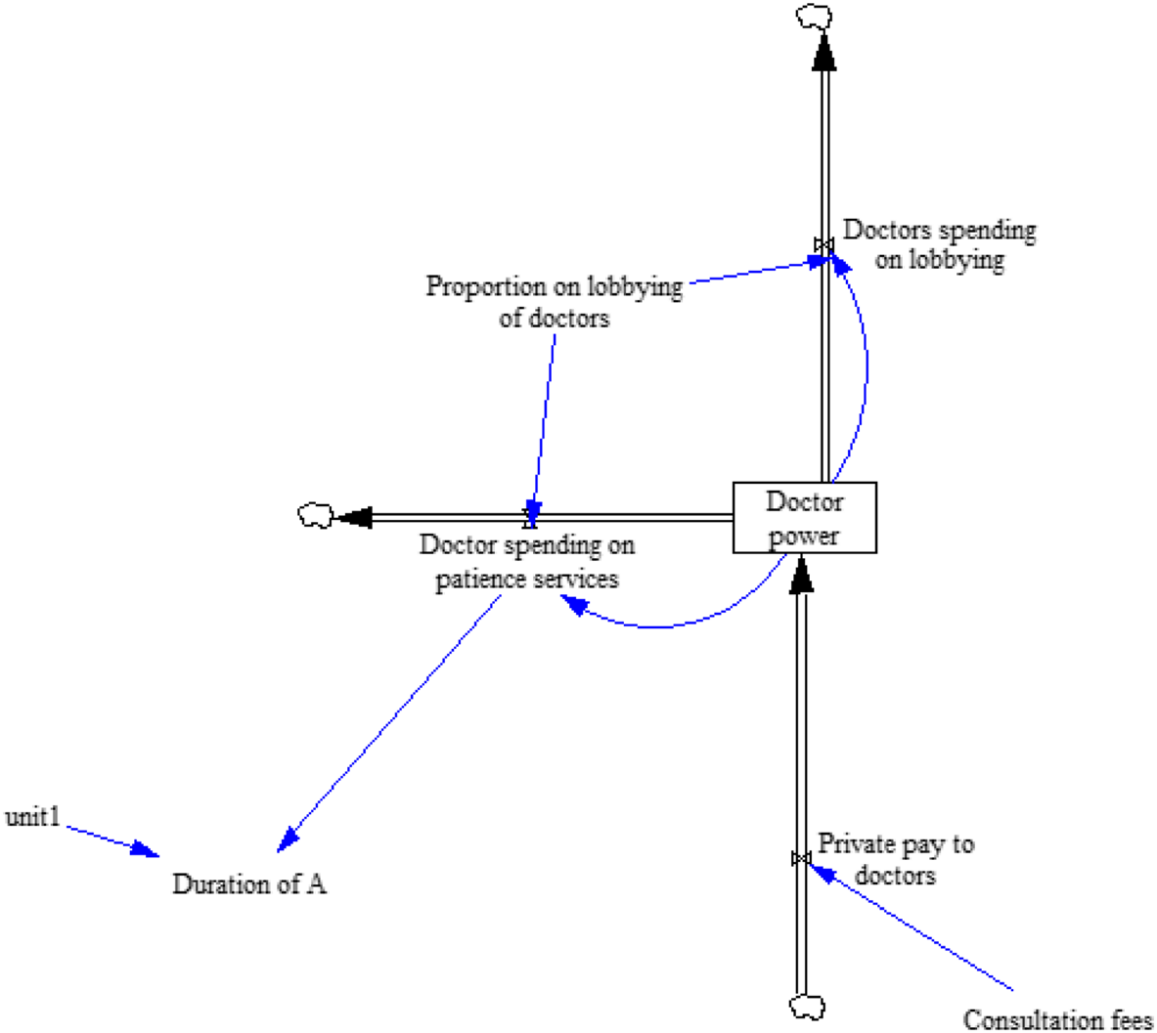
**Subsystem 3: lobbying process – doctor power can be depleted by spending either on providing patient services or on lobbying for more resources.** The proportion of power spent on lobbying is a constant fraction in each run of the model. The more doctors spend on patient services the shorter the duration of disease A for patients who contract that disease.

**Figure 4:**
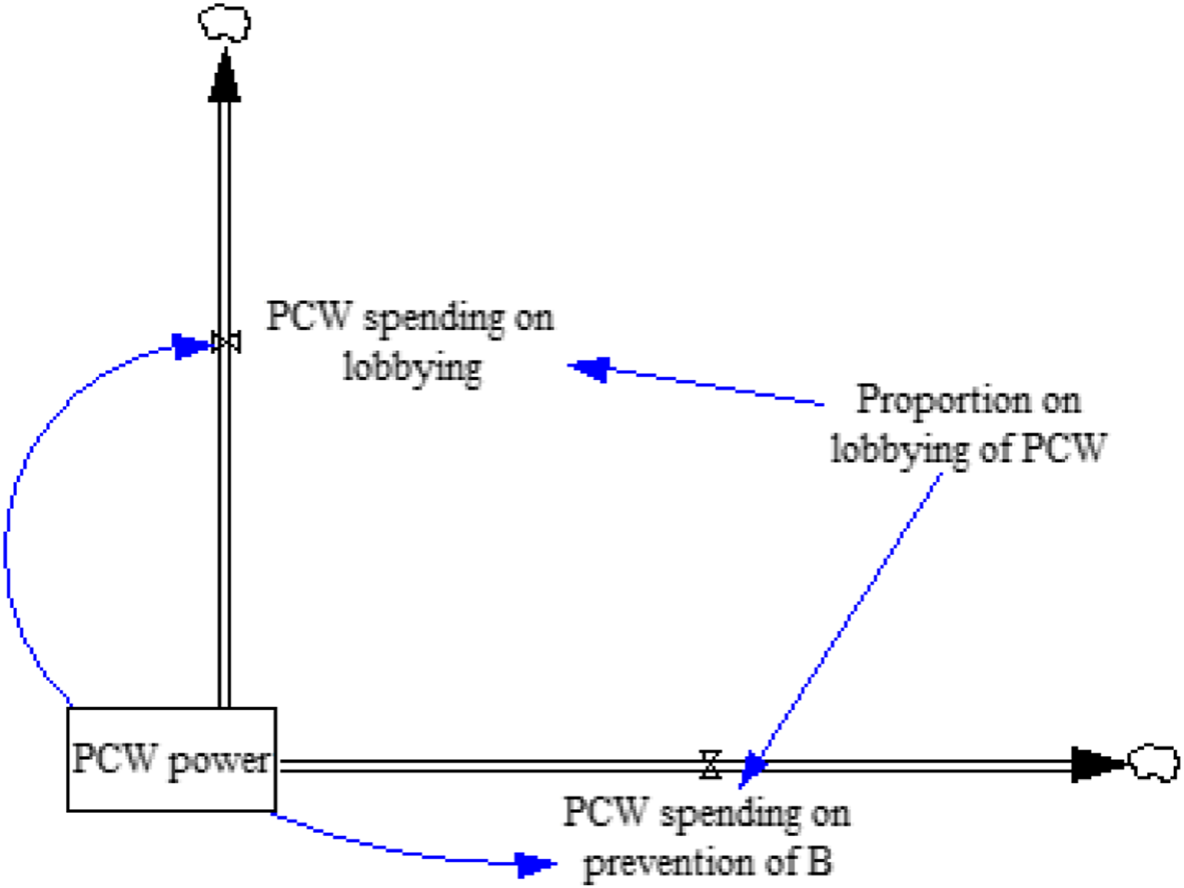
**Subsystem 3: lobbying process – PCW power is spent on lobbying and on shortening the incidence of death from disease B.** The proportion of power spent on lobbying is a constant fraction in each run of the model.

Figures 3 and 4 illustrate the final subsystem in the model – the lobbying process driven by doctors and PCWs. Table 3 presents the parameters, their units, and any assumptions that are relevant. We define political bargaining power of doctors as proportional to resources collected in a common lobbying fund by doctors out of their public and private salaries. The doctors, as a group, can then allocate the money they earn from fees, government payments, and donor payments to either improving recovery time for disease A in the infected population or to lobbying activity so they can capture more public funds. Similarly, we define PCW power as the resources amassed by PCWs from all sources, which can then be allocated to preventive services or lobbying. As mentioned above, PCW resources include public funds and donor funding, but there are no fees paid for PCWs.

**Figure 5:**
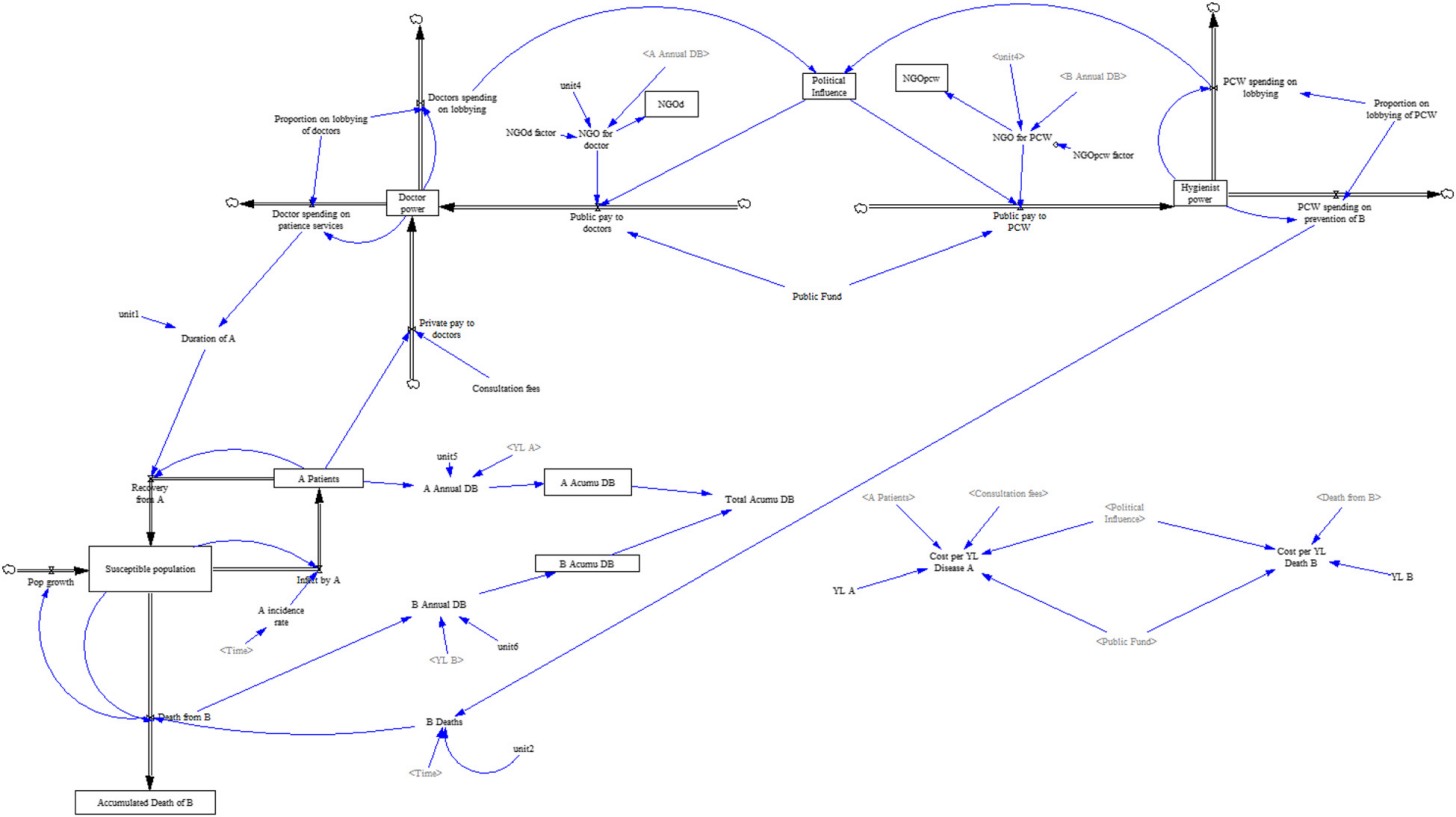
**System dynamic model showing the factors affecting resource allocation.** This figure displays how all of the previously introduced sub-systems relate.

 The entire model, showing how the sub-systems relate, is displayed in Figure 5.

**Table 3:** Subsystem 3 variables and assumptions

**Equilibrium parameters**	**Variable type**	**Initial value**	**Notes and assumptions**
Doctor power	State	1,000	Doctor’s lobbying power at equilibrium; equals PCW power at equilibrium
Doctor spending on lobbying	Rate	0	Doctor power × Proportion on lobbying
Proportion on lobbying of doctors	Auxiliary	0.125	Doctors spent 12.5% of their resources on lobbying
Doctor spending on patient services	Rate	0	Doctor power × (1–Proportion on lobbying of doctors)
PCW power	State	1,000	PCW’s lobbying power at equilibrium; PCWs and doctors start with the same level of power
PCW spending on lobbying	Rate	0	PCW power × Proportion on lobbying of PCWs
Proportion on lobbying of PCWs	Auxiliary	0.5	PCWs spend 50% of their resources on lobbying
PCWs spending on prevention of disease B	Rate	0	PCW power × (1-Proportion on lobbying of PCWs)

#### Modeling scenarios

The model horizon is 200 months. During both modeling and sensitivity analyses, we tracked changes in cumulative disease burden and deaths from both diseases and cumulative spending. We used the cumulative burden of disease and the cumulative spending to produce the graphs of costs vs. DALYs averted by the various donor spending policies.

A standard run with the simulation requires observing its response to two types of simulated shocks: disease epidemics and external funding from donors. Without shocks, the model stays in perpetual equilibrium. Epidemic times for disease A were arbitrarily selected to fall at months 13, 49, 85, and 121; each epidemic lasted one year. During an epidemic of disease A, the incidence rate increased ten-fold, from 0.05 to 0.5. Epidemic times for disease B were selected to fall at months 37, 61, 109, and 121. We tried different epidemic combinations and timings. Specifically, the system encountered an epidemic of A alone, then an epidemic of B alone, then two epidemics of A followed by B, and, finally, an epidemic of A and B together. We examined the system’s ability to contain these epidemics with and without the response of NGO donors.

**Figure 6:**
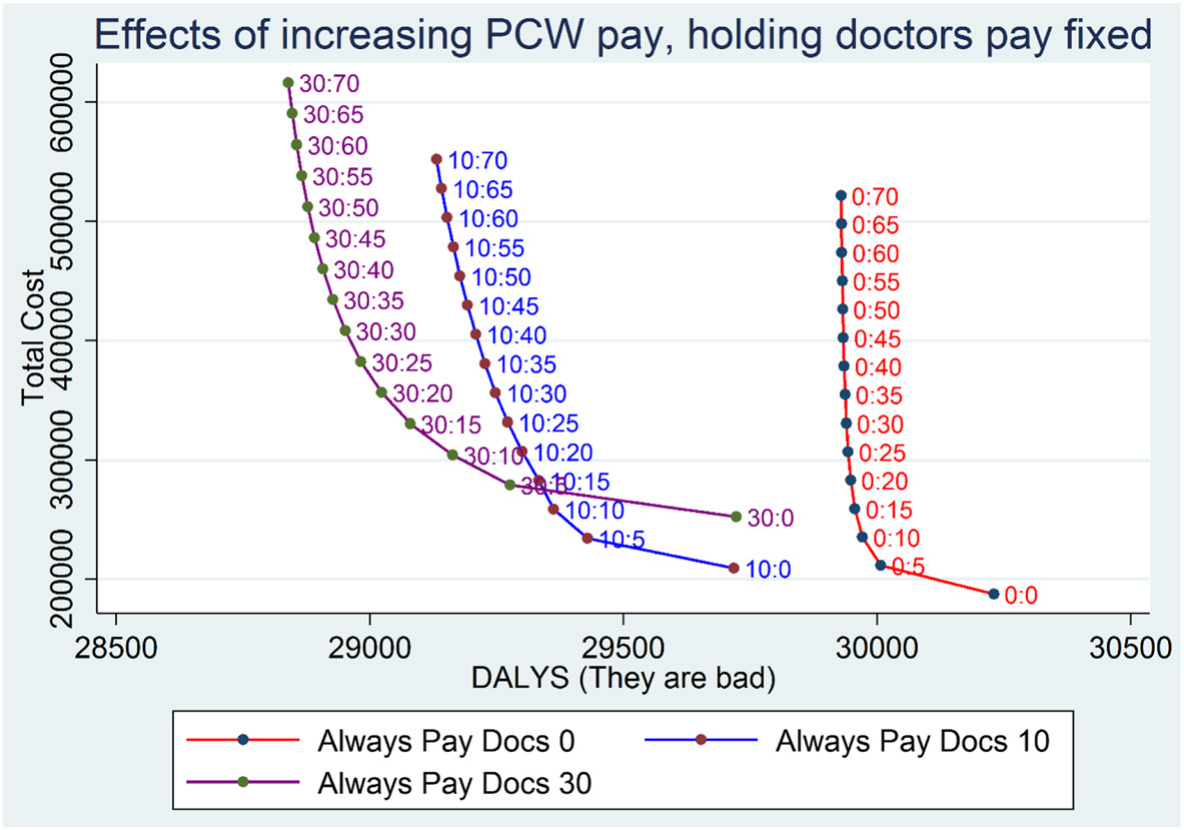
**Baseline scenario: results of NGO donation policies at various fixed levels of NGO donation per epidemic case of disease A from 0 to 30.** Points are labeled with D_A_:D_B_, which represent, respectively, the $ per additional DALY of disease A and of disease B. At all additional payments to the prevention workers, paying more reduces the burden of disease.

**Figure 7:**
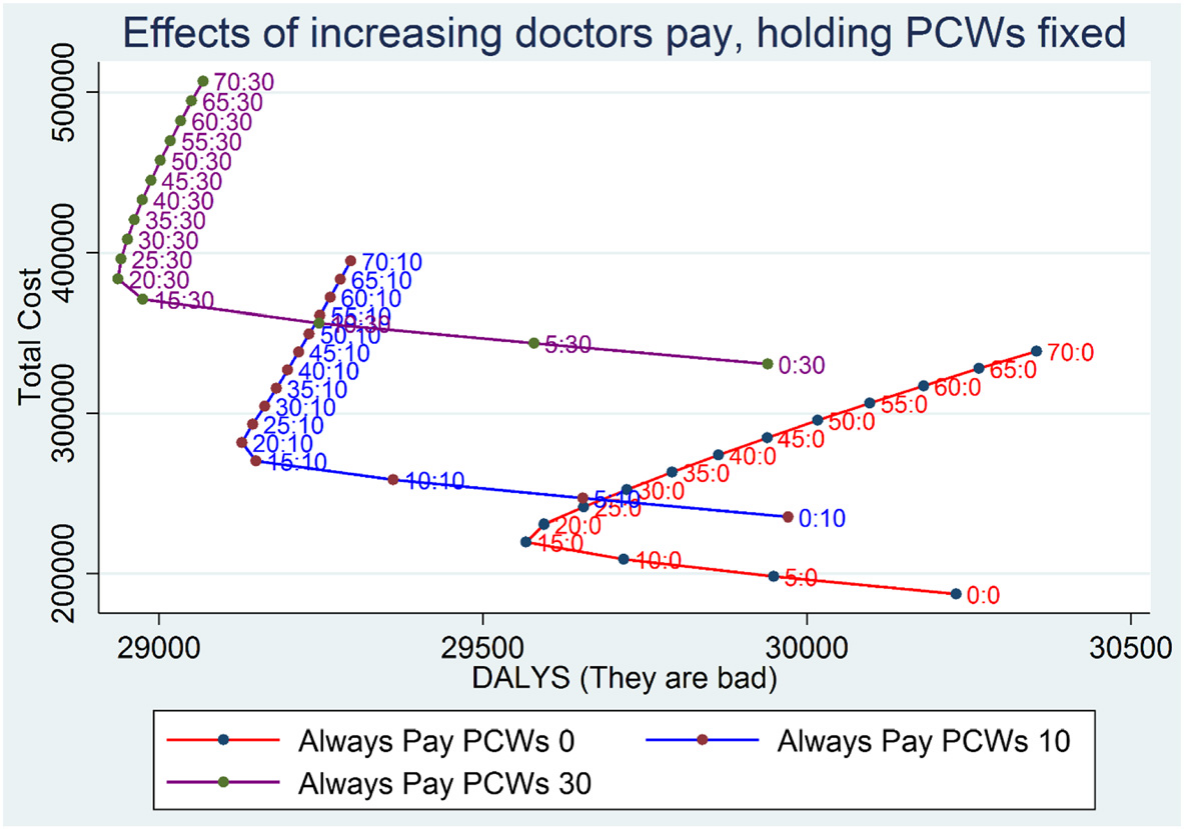
**Baseline scenario: evidence of population harm from expanding payments to doctors.** The parts of the cost effectiveness curves sloping up and to the right indicate options that cost progressively more and increase the burden of disease. These undesirable options occur beyond a threshold of $20 per DALY of disease A. Points are labeled with D_A_:D_B_, which represent, respectively, the $ per additional DALY of disease A and of disease B. D_B_ is held constant within each iso-policy curve.

NGO donors are programmed to make donations to their chosen disease only as long as the incidence rate is higher than the equilibrium baseline level. Donation policies are specified as the number of additional dollars allocated to either disease per DALY averted. D_A_ was defined as the donation in terms of $ per additional DALY of disease A after an epidemic of A and D_B_ as the donation in terms of $ per additional DALY of disease B that the NGO would spend. Suppose the donation policy is D_A_ = $10 per epidemic DALY of A and $D_B_ = 5 per epidemic DALY of B. In an epidemic with 10 additional DALYs of A and 6 additional DALYs of B, the donations would be $100 to A and $30 to B. In equilibrium, D_A_ was set to equal D_B_. There was no built-in role for donors to practice disease exceptionalism [26]. To test the effects of special pleading on behalf of either disease, we later let donation policies become discordant and we tested donation policies with D_A_ set sequentially at {0, 5, 10, 20, 30, 40, 50, 60, 70} while, independently, D_B_ realized values from {20, 30, 40, 50, 60, 70} [27]. A donor whose policy was D_A_ = 70 and D_B_ = 10 would be indicating a bias to spend seven times as much averting a DALY from disease A relative to disease B. A total of 81 (D_A_, D_B_) pairs were tested and we tracked the 200 month cumulative sum of DALYS from both of the diseases and total costs to the government, patients, and NGOs as a result of the NGO’s epidemic control strategies. Overall, 81 cycles were required through the 200 month trajectory of the model to fully assess a policy or parameter change. The graphical output of these policy assessments involves showing a four-dimensional manifold of costs and DALYS as co-determined outcomes of D_A_ and D_B_. We accomplished this by showing isoquants – a locus of DALY and cost outcomes generated when D_A_ (or D_B_) is held constant while D_B_ (or D_A_) varies along the locus (Figures 6 and 7).

The economic problem for the NGO ought to be choosing epidemic response policy (D_A_, D_B_) to minimize DALYs while minimizing total cost to itself and to society. Whether or not it espouses an economic paradigm, the NGO does not want to achieve a situation where they spend more and achieve more overall deaths and DALYs due to their spending crowding out other activities in the health system. Locating attractive and unattractive values of (D_A_, D_B_) is our schematic version of why donors care about data on the global burden of disease and why they want data on the effectiveness and cost-effectiveness of health interventions.

#### Sensitivity analyses

We conducted the following sensitivity analyses in order to ensure that the results we obtained from the main model were robust to changes in the original parameters and variables:

•We let years of life lost from disability per case of disease A vary ±10% from 0.45, 0.5, and 0.55.

•We let years of life lost from death per case of disease B vary ±10% from 22.5, 25, and 27.5.

#### Policy analyses

We implemented three different policy scenarios to discover how various strategies might make the system work better.

•Eliminate lobbying for curative care and preventive care.

•Making the public pay responsive to burden of disease changes resulting from epidemics.

•Removing the consultation fee obtained by doctors.

## Results

We found that several negative feedback loops stabilized key levels in the model. There was negative feedback restoring physician incomes to baseline after their incomes rose in an epidemic. Essentially, the epidemic gave the doctors more money, but they ultimately spent some of the money reducing the size of the caseload that generated their revenue and eventually their revenue fell. The burden of disease A was also restored to equilibrium through this negative feedback loop.

The model also showed the presence of a negative feedback loop for government to respond to epidemics of disease B with extra spending on disease B. After disease B returned to baseline, the rational government response to the epidemic stopped. In the absence of the government response to epidemics of disease B, there is no natural way the system would raise funding for PCWs to fight an epidemic of disease B and the system would simply shift to a higher disease burden and not show return to equilibrium.Figures 6 and 7 plot societal dollars spent vs. DALYs averted. Note that the optimal zone in these figures would be at the bottom left where nothing is spent and there is no DALY burden. Points that are at the far right represent high disease burden and little spending. Starting from the bottom, as more is spent there is a rise in the vertical dimension and the DALY burden can become lower, moving to the left. As shown in Figures 6 and 7, there were diminishing returns to scale from NGOs investing in either disease – progressive amounts of money spent in the vertical dimension led to smaller and smaller reductions in DALY burden. Figure 6 shows that, with spending on doctors held fixed, more spent on PCWs had a continuously increasing incremental cost per DALY averted.

Surprisingly, Figure 7 shows that spending on doctors generated negative returns with overall declines in population health per incremental $ spent on doctors’ services per epidemic DALY of disease A after spending surpassed a threshold. The harm is exclusively due to a higher DALY burden of unprevented cases of disease B. These cases of B were not prevented because the windfall payments to doctors during an epidemic of disease A were used to lobby the government for a larger share of the fixed health budget. An epidemic of disease A would trigger more fee based revenue for doctors, more NGO based revenue for doctors, and more government based revenue as a rational response. Whereas the NGOs and government were obligated to allocate funds rationally in proportion to the DALY burden, the sick patients, during epidemics of disease A, were not pursuing an optimal societal allocation of revenue to doctors. The patient revenue to doctors helped their lobbying power grow abnormally large. Patient-driven revenue led to so much doctor lobbying power that the prevention budget became squeezed towards zero and the burden of disease B rose. In models where D_A_ was a lot higher than D_B_, the iatrogenic epidemic of disease B could trigger remedial spending by the donor, but government health budgets had limited response to the epidemic due to doctor lobbying. Spending on doctors during the epidemic of disease A leads to doctors’ lobbying activity depleting the fixed government allocations to PCWs and leaving the population vulnerable to disease B. Without the agency of PCWs to prevent disease B, people perished in higher numbers. In Figure 7, the model shows negative returns to investment in responding to cures with an inflection point occurring around $20 per DALY of disease A. Being willing to spend more than $20 per case of disease A in an epidemic of disease A creates a higher death rate from unprevented cases of disease B.

Our sensitivity analysis, which focused on testing different DALY weights for diseases A and B as well as disruptions in funding to either doctors or PCWs, confirmed the robustness of the phenomenon of negative returns to spending high amounts per case of B. We observed the same findings despite 10% shifts in the DALY weight for either disease and despite 10% shifts in the government’s standard response to epidemics, signaling that our conclusions were not simply a product of our initial set of parameters.Policy analysis explored what happens in a context where doctors lose their ability to earn consultation fees from seeing patients. By removing doctors’ disease-driven earning, and therefore making their income symmetrical to PCWs, we saw the backward bending cost-DALYs effects observed in Figure 7 disappear. Another policy analysis removed all lobbying power of both doctors and PCWs. By removing lobbying from our model, we no longer observed the backward bending effects seen in Figure 7. Either modification was enough to eliminate the damaging effects on population health of spending more money on curative services. Both solutions were effective and the results qualitatively resemble Figure 7 (results available in a supplementary appendix).

## Discussion

This system dynamics model of resource allocation explicitly models political influences on the allocation of public spending in the health sector. It illustrates potential unintended consequences from fully rational NGO contributions to respond to epidemics. Considerations from complex adaptive systems guided the exploration of non-linear, unintended consequences in a simulated health system – something that cannot be accomplished using traditional cost-effectiveness analysis techniques. The results offer several cautions about irrational distortions that occur in human systems even when rational policies are pursued. The donors in the simulation were efficiently allocating resources in proportion to the opportunity to avert the most DALYs per dollar, but political lobbying resulted in their allocations to the doctors snowballing into a large political lobbying fund that starved the preventive work of the PCWs and led to higher costs and unnecessary deaths.

It is significant to note that the NGO’s response to epidemics of disease A did not factor in the fungibility of private spending on disease A. The NGO was programmed to assume that its donations were not being matched by private sector patient payments. If the NGO had been reducing its donations dollar for dollar in response to private spending, then the tragic loss of life from the crowd out of preventive spending would not have occurred. Traditional cost-effectiveness models have no way to simulate this type of unintended consequence because traditional cost-effectiveness measures costs, but says little about who will ultimately pay the cost. Furthermore, traditional cost-effectiveness cannot accommodate a positive feedback loop in which spending on a particular health resource creates obligations to irrationally escalate spending on that resource due to political pressure from those who prosper from the initial allocation.

Our findings add further insights into the debate on optimal resource allocation between curative and preventive care. Because curative medicine offers services whose benefits are privately enjoyed by each sick person they are defined by economics as “excludable goods”. Market mechanisms work well to balance supply of resource flows in proportion to the demand for excludable goods [28]. In contrast, the benefits of community prevention-work are non-excludable; everyone benefits but wants someone else to pay. With no defined property rights to transmit an effective demand signal to suppliers, the system is prey to failure and mal-adaptation. The under-investment in community prevention joins a long list of many other market failures in health [28]. Community health advocacy falls to an assorted collection of community advocates, academics, and enlightened spokespersons [29]. In public policy-making it is common to hear advocates declare that their sector is “high priority” and requires special treatment [30]; it can be difficult at times to assess the legitimacy of competing claims. However, the missing market for population-level prevention efforts and their history of extremely large benefits at low cost suggests that this might be an area that is perennially and inappropriately neglected.

Our model’s focus on the asymmetric ability of the doctor class to achieve political clout from billable fees offers a cautionary lesson for NGO donors. When donors compassionately invest in curative strategies they may unwittingly contribute to the rent-seeking capability of the doctors. Health systems can inappropriately de-prioritize prevention efforts delivered at population level. If it were easy to do so, an NGO or donor should stay informed about private revenue streams that could be augmenting their own allocation to address health problems. These private revenue flows can unbalance what could have been a measured response to a health issue, and require the donor to offset its contribution accordingly or focus to sub-populations that were not being covered privately.

Funding for HIV/AIDS treatment programs in low-income countries may be an example where cure is crowding out prevention through a mechanism similar to the one in the model. Countries with limited human resources for health cannot absorb large amounts of donor spending on antiretroviral programs without staff redeploying away from their primary care jobs into the well-funded antiretroviral programs. Evidence of weaker performance on maternal and child health programs where HIV burdens are higher might be suggesting this sort of tradeoff [31,32].

Although we use the word “lobbying”, the basic dynamics observed in the model do not require there to be an organized medical, pharmaceutical, or hospital association with a specific lobbying fund supporting politicians; real-world health care interest lobbies are much more subtle. Health care lobbying can be emotionally appealing and seductive. It is common for motivated disease advocates to spotlight a photo with a victim of a curable disease, or to note how a disease victim’s treatment is a human right [33,34]. Global donors are emphasizing disease-curing funds (e.g., providing AIDS, tuberculosis, and malaria treatments). The drive towards universal health insurance coverage is also an instance of lobbying that serves clinical interests while addressing rights to health care services. The growth of health insurance can trigger a self-perpetuating feedback loop of political power by those who provide clinical care. One approach to protect prevention would be to ring fence prevention funds. Another approach would allocate donor funds preferentially to the neglected prevention sector as was done during a World Bank loan to Argentina [35]. Both of these approaches face challenges if the government is under high lobbying pressure. The ring fence can be punctured and the aid can be fungibly reallocated unless there is high commitment by the government to preserve a high priority sector [30].

Our model shows that the harmful spiral can be eliminated by eliminating patient fee payments or by eliminating any lobby-type linkage between health worker revenue and government spending. Although these extreme scenarios were modeled, total elimination is impractical in the real world. The reason to focus on patient fees is that it is only the fees that give doctors asymmetric growth in epidemic-driven revenue that is not enjoyed by PCWs. Epidemics of disease A enriched the doctors in payments from both doctors and the government. Epidemics of disease B enriched the PCWs only from government or donor funds because there could be no private market based on preventing disease B. The absence of a functioning market for community prevention efforts requires a central planning function to step into the breach and dial up or down government spending based on epidemiological data and data on total health expenditure. Our paper emphasizes that this central intervention is prone to being captured and manipulated by selfish interest groups and requires institutional measures that insulate it. The National Health Service of the UK offers an example of a policy solution to this dilemma in which public health budgets are “ring-fenced” to prevent clinical enterprises from infringing on them [36].

Our model provides a quantitative application of complex adaptive system methodologies to health care systems and policy analysis [37-40]. It is an illustrative tool which was created to foster insight and understanding of unintended consequences from the financing of curative and prevention care. Preventive community-wide efforts in health labor under several disadvantages. When preventive services need to compete against cure for a common pool of government resources they are likely to suffer. They can be politically outgunned because curative health workers have the ability to amass more money through fees while community prevention workers cannot. A zero-sum game played between prevention and cure is not a fair contest.

## Abbreviations

DALYs: Disability-adjusted life years; NGO: Non-governmental organization; PCWs: Preventive care workers.

## Competing interests

The authors declare that they have no competing interests.

## Authors’ contributions

DB led the conceptualization and implementation of this activity. DB, LP, and QL developed the final model and analyzes, as well as prepared the first draft of the manuscript. DHP and AAH contributed to revisions and finalizing the manuscript. All authors read and approved the final manuscript.
